# Supplementary material for: Educational video combined with augmented clinical support to improve CPAP use in patients with obstructive sleep apnea-hypopnea syndrome: A randomized controlled trial protocol
Source: PLoS One. 2025 May 30;20(5):e0322285. doi: 10.1371/journal.pone.0322285 (PMC12124739; doi:10.1371/journal.pone.0322285)
Supplement: S3 File — Video 1: Be khayr – بخير. (Dec 15, 2024). إعلان فيلم ليلتك زينة – Offical trailer Liltek Zina l. https://www.youtube.com/watch?v=qzzCzxLSJts. Video 2: Be khayr – بخير. (Apr 3, 2025). AGENDA_SAS. https://www.youtube.com/watch?v=iirm-0Oi6YU&t=1s. (DOCX) [file pone.0322285.s003.docx]

Please find the **link** to Video 1 attached:

<https://www.youtube.com/watch?v=qzzCzxLSJts>

Please find the **link** to Video 2 attached:

<https://www.youtube.com/watch?v=iirm-0Oi6YU>
