# Supplementary material for: Educational video combined with augmented clinical support to improve CPAP use in patients with obstructive sleep apnea-hypopnea syndrome: A randomized controlled trial protocol
Source: PLoS One. 2025 May 30;20(5):e0322285. doi: 10.1371/journal.pone.0322285 (PMC12124739; doi:10.1371/journal.pone.0322285)
Supplement: Attachment — Submitted filename: Reponses to the Editor.docx [file pone.0322285.s004.docx]

Dear Sir Edwin Ularte,

Thank you for the opportunity to improve our manuscript. We are resubmitting our manuscript entitled “Educational Video Combined with Augmented Clinical Support to Improve CPAP Use in Patients with Obstructive Sleep Apnea-Hypopnea Syndrome: A Randomized Controlled Trial” [EMID: 56ac9f32a557e898], after addressing the requested edits. All modifications in the manuscript and on the title page have been highlighted in yellow for your convenience.

1. PLOS ONE requires that all clinical trials are registered in an appropriate registry (the WHO list of approved registries is at https://www.who.int/clinical-trials-registry-platform/network/primary-registries" https://www.who.int/clinical-trials-registry-platform/network/primary-registries and more information on trial registration is at http://www.icmje.org/about-icmje/faqs/clinical-trials-registration/).

   Please state the name of the registry and the registration number (e.g. ISRCTN or ClinicalTrials.gov) in the submission data and on the title page of your manuscript.
   a) Please provide the complete date range for participant recruitment and follow-up in the methods section of your manuscript.

b) If you have not yet registered your trial in an appropriate registry, we now require you to do so and will need confirmation of the trial registry number before we can pass your paper to the next stage of review. Please include in the Methods section of your paper your reasons for not registering this study before enrolment of participants started. Please confirm that all related trials are registered by stating: “The authors confirm that all ongoing and related trials for this drug/intervention are registered”.

Please see http://journals.plos.org/plosone/s/submission-guidelines#loc-clinical-trials for our policies on clinical trials.

**RESPONSE 1**

Our clinical trial was registered in the Pan African Clinical Trial Registry (pactr.samrc.ac.za) database.

Our unique identification number for the registry is **PACTR202412813486845**.

The name of the registry and the registration number (e.g. ISRCTN or ClinicalTrials.gov) were stated in the submission data and on the title page of our manuscript.

All patients referred to the Department of Respiratory and Sleep Medicine at Hedi Chaker University Hospital of Sfax-Tunisia, for suspected OSAHS will be screened for eligibility **between January 2025 and July 2025.** The study will be **terminated** by **December** 31^st^ 2025.

**2**. Please note that you must upload a completed CONSORT checklist as a supporting information file. Blank copies of this document and information regarding CONSORT can be found via the following link: (https://www.equator-network.org/reporting-guidelines/consort/). If your clinical trial uses a non-randomized design, you may wish to submit a TREND checklist (http://www.cdc.gov/trendstatement), in place of the CONSORT checklist.

**RESPONSE 2**

The CONSORT checklist was completed.

**3.** Please include in your methods section, and in the ‘ethics’ field in the submission system (via Edit Submission), the name(s) of the ethics committee(s)/institutional review board(s) (IRBs) that approved your study. If your study was conducted at multiple institutions/hospitals/centers, provide the name of each committee that considered and approved your research. If you did not obtain ethics approval for your study, please explain in your manuscript why ethics approval was not necessary.

**RESPONSE 3**

In our methods section, and the ‘ethics’ field in the submission system, we included the name of the ethics committee that approved our study [Ethics Committee of the Hedi Chaker University Hospital of Sfax (Tunisia)].

Best regard

Sameh MSAAD, MD

The University of Sfax, Faculty of Medicine of Sfax, Sfax-Tunisia
