# Supplementary material for: Educational video combined with augmented clinical support to improve CPAP use in patients with obstructive sleep apnea-hypopnea syndrome: A randomized controlled trial protocol
Source: PLoS One. 2025 May 30;20(5):e0322285. doi: 10.1371/journal.pone.0322285 (PMC12124739; doi:10.1371/journal.pone.0322285)
Supplement: Attachment — Submitted filename: Response to Reviewers.docx [file pone.0322285.s005.docx]

February 16, 2025

**Manuscript-Resubmission**: [**Educational video combined with augmented clinical support to improve CPAP use in patients with obstructive sleep apnea-hypopnea syndrome: a randomized controlled trial Protocol**].

**Manuscript Number:** PONE-D-24-46553

**Academic Editor PLOS ONE:** Yongzhong Guo, Ph.D

Dear Editor and Reviewers,

Thank you for your insightful comments. On behalf of my co-authors, I sincerely appreciate the time and effort you have dedicated to reviewing our manuscript titled "Educational Video Combined with Augmented Clinical Support to Improve CPAP Use in Patients with Obstructive Sleep Apnea-Hypopnea Syndrome: A Randomized Controlled Trial Protocol."

Your feedback has been invaluable. We have carefully considered your suggestions and revised the manuscript accordingly. Our responses to your comments are provided in a point-by-point format below. Additionally, we have submitted a revised version of the manuscript, with changes highlighted in yellow.

We look forward to your response regarding our revisions and are more than willing to make any further changes that would enhance the paper. Thank you once again for your attention and consideration.

Best regards,

Sameh MSAAD, MD

Faculty of Medicine. University of Sfax-Tunisia

**Journal requirements**

When submitting your revision, we need you to address these additional requirements.

**1**. Please ensure that your manuscript meets PLOS ONE's style requirements, including those for file naming. The PLOS ONE style templates can be found at

<https://journals.plos.org/plosone/s/file?id=wjVg/PLOSOne_formatting_sample_main_body.pdf> and <https://journals.plos.org/plosone/s/file?id=ba62/PLOSOne_formatting_sample_title_authors_affiliations.pdf>

**Response:** As you recommended, we have carefully checked that our manuscript meets PLOSE ONE’s requirements. References were presented in Vancouver style.

**2**. In the Methods section of your revised manuscript, please include the full name of the institutional review board or ethics committee that approved the protocol, the approval or permit number that was issued, and the date that approval was granted.

**Response:** We apologize for this oversight. All necessary information regarding ethical approval was included in the revised manuscript**.**

**3**. Please include “Protocol” in the manuscript title.

**Response:** As you suggested, we included “Protocol” in the manuscript title.

4. We note that the grant information you provided in the ‘Funding Information’ and ‘Financial Disclosure’ sections do not match. When you resubmit, please ensure that you provide the correct grant numbers for the awards you received for your study in the ‘Funding Information’ section.

**Response:** We sincerely apologize for this oversight. We have thoroughly reviewed and rectified the information regarding the funding of our protocol.

**Reviewers' comments**

**Reviewer #1**

1. Please number the references and list them after the conclusion in the order they appear in the manuscript.

**Response:** Thank you for your comment. Following your suggestion, references have been organized according to the Vancouver style as outlined in the guidelines of PLOS ONE**.**

2. Please mention the entire process including data collection and intervention in the past tense.

**Response:** At the time of protocol drafting, data collection and intervention had not yet begun. Therefore, we describe the entire process in the future tense.

3. A 1-week data might be too little for changing auto CPAP to fixed CPAP. Have compliance, residual AHIs, leaks, etc accounted for before switching to fixed CPAP?

**Response:** Response: We agree with this assessment; therefore, we have decided to extend the Auto-CPAP titration duration to two weeks instead of one.

4. While nasal obstruction is a common symptom and a risk factor, snoring is even more commonly present in OSA. Has snoring been assessed?

**Response:** Thank you for your interesting suggestion. We have added a section for assessing snoring. Snoring will be evaluated using the question, "Do you snore?" with the following response options: "never," "rarely," "sometimes," "often," and "almost always." Based on these responses, participants will be categorized into three groups: (1) "Never" or "Rarely," (2) "Sometimes," and (3) "Often" or "Almost Always."

5. While the methods have been described, I do not find the results of the study anywhere in the manuscript.

**Response:** At the time the protocol was drafted, data collection and intervention had not yet started. Therefore, we do not have any results at this point.

6. The manuscript cannot be assessed without complete data.

**Response:** This is a protocol study scheduled to take place from January 2025 to July 2025, with a planned conclusion by December 31, 2025. As a result, we do not have any results available at this time. We look forward to sharing our findings with you in our upcoming paper.

**Reviewer #2**

Overall, the study protocol looks good. It would be great to have real data. It may bring more meaningful outcomes to this important field.

**Response:** Thank you for your valuable comment and suggestion. We would like to inform you that this protocol study is scheduled to occur from January 2025 to July 2025, with a planned conclusion by December 31, 2025. Consequently, we do not have any results to share at this moment. We look forward to presenting our findings in our forthcoming paper.

**Reviewer #3**

Here is a list of specific comments.

Note: line numbering is not available, and page numbering in reviews
and comments is based on ruler applied in Editorial Manager-generated PDF.

**1**. I did not find the CONSORT checklist.

**Response:** Thank you for your remark. the CONSORT checklist has been already submitted to the journal.

**2**. I did not find the study protocol as a supplement.

**Response:** Thank you for your remark. The study protocol as a supplement has been already submitted to the journal.

**3**. Page 6, Section 2.3.4, line 1: I suggest removing “unadjusted.” Adjusting or not would be part of the statistical analysis plan, and does not affect the definition of the outcome.

**Response:** We appreciate your input and have followed your recommendation by removing the term “unadjusted” in Section 2.3.4.

**4.** Page 6, Section 2.3.4.1, lines 8–10: Should “good adherence” at 3 months be one of the outcomes?

**Response:** We consider "good adherence" at 3 months to be a primary outcome of our study. This data is mentioned more clearly in the manuscript.

**5.** Page 6, Section 2.3.4.1, lines 11–13: Should “patients’ perception of CPAP” be one of the outcomes? It might not be any difference between groups because the questionnaire was administered after the
the first night of CPAP titration. Was the questionnaire administered again later (e.g., 1, 3, or 6 months)?
What was the reason for administering the questionnaire? Was the questionnaire used to tailor the
EPT workshops?

**Response:** Thank you for highlighting the need for clearer details. The Sleep Disorders Center CPAP Questionnaire (SDC CPAP Questionnaire) will be administered only once, on the morning following the first night of CPAP titration. The responses to this questionnaire can predict how well a patient will adhere to CPAP therapy during the first 30 days. This information allows clinicians to identify patients at risk of non-adherence to treatment. The SDC CPAP Questionnaire will be administered by the nurse for each patient in the PET group on day 2 following CPAP initiation. We will use the mean CPAP perception score to create 2 categories of patients: poor CPAP perception (scores > 16) and good CPAP perception (scores ≤ 16). We hypothesize that a poor CPAP perception score would independently predict poor CPAP adherence during the first 30 days of therapy) (Balachandran et al., 2013). For patients in the TPE group with a poor CPAP perception score, a supplementary follow-up appointment with a sleep physician will be scheduled for day 15 after CPAP initiation.

**6.** Page 8, Section 2.4.1, line 1: It was not clear how the statistical analysis in section 2.7 can answer “optimal adherence to CPAP.”

**Response: In section 2.3.4.1,** Good adherence to CPAP at 3 months was defined as a CPAP use for ≥4 h per night on ≥ 70% of nights within a consecutive thirty-day period anytime during the last 90 days of therapy.

**7.** Page 8, Section 2.4.1, line 5: I suggest spelling out EPT at its first appearance.

**Response:** Thank you for your suggestion. The abbreviation (TPE) of Therapeutic patient education was spelt out at its first appearance in the background [Over the last two decades, several interventions have been proven to increase CPAP adherence, including technological advances in CPAP devices, augmented clinical support **(ACS)** (more frequent appointments, telephone calls, home visits), therapeutic patient education **(TPE)**, cognitive behavioral therapy and motivational enhancement. There is an increasing tendency for most clinicians to offer their patients a combination of ACS and TPE].

**8.** Page 9, Section 2.4.2, lines 1–2: The sentence appeared twice within few lines on this page. I suggest
reducing the redundant words as much as possible.

**Response:** We apologize for the redundancy and have removed it, as noted in the manuscript. In the paragraph just before section 2.4.4, we referred to the educational team without providing details. Information regarding the educational team was mentioned exclusively in section 2.4.2**.**

**9.** Page 9, Section 2.4.5: I suggest calling out Table 1 in Section 2.4.5. The previous calling out in
Section 2.4.1 may not be ideal.

**Response:** As you suggested, table 1 was called out in Section 2.4.5. instead of section 2.4.1.

**10.** Page 10, Section 2.6, lines 21–22: This answered Comment 5 above. I suggest revising the earlier
sentence where Comment 5 referred to.

**Response:** Section 2.6 and Figure 1 have been revised as you suggested. All details are available in response to comment 5.

**11.** Page 11, Section 2.7: Were there sample size and power calculation to determine the required sample
sizes per group?

**Response:** We did not calculate the sample size because we consider this a pilot study**.**

**12.** Page 11, Section 2.7, line 7: The intervention had two groups. I suggest clarifying what “3 or more
groups” referred to.

**Response:** The ANOVA test will be used for comparing two variables with three categories each.

**13.** Table 1: There were only two timestamps, reception and return. I suggest adding a timestamp for each
row. Also, please check the return home timestamp. Was 12 AM correct?

**Response:** Thank you for this interesting suggestion. As you recommended, we have added a timestamp for each row. We also corrected the return time: it is 12:00 pm and not 12:00 am.
